# Performance Analysis of Wirelessly Powered Cognitive Radio Network with Statistical CSI and Random Mobility

**DOI:** 10.3390/s23094518

**Published:** 2023-05-06

**Authors:** Nadica Kozić, Vesna Blagojević, Aleksandra Cvetković, Predrag Ivaniš

**Affiliations:** 1 School of Electrical Engineering, University of Belgrade, 11000 Belgrade, Serbia; kn155029p@student.etf.bg.ac.rs (N.K.); predrag.ivanis@etf.rs (P.I.); 2 Faculty of Mechanical Engineering, University of Nis, 18000 Nis, Serbia; aleksandra.cvetkovic@masfak.ni.ac.rs

**Keywords:** cognitive radio, ergodic capacity, Internet of Things, outage probability, power beacon, random waypoint mobility model, spectrum sharing, statistical channel state information, underlay, wireless power transfer

## Abstract

The relentless expansion of communications services and applications in 5G networks and their further projected growth bring the challenge of necessary spectrum scarcity, a challenge which might be overcome using the concept of cognitive radio. Furthermore, an extremely high number of low-power devices are introduced by the concept of the Internet of Things (IoT), which also requires efficient energy usage and practically applicable device powering. Motivated by these facts, in this paper, we analyze a wirelessly powered underlay cognitive system based on a realistic case in which statistical channel state information (CSI) is available. In the system considered, the primary and the cognitive networks share the same spectrum band under the constraint of an interference threshold and a maximal tolerable outage permitted by the primary user. To adopt the system model in realistic IoT application scenarios in which network nodes are mobile, we consider the randomly moving cognitive user receiver. For the analyzed system, we derive the closed-form expressions for the outage probability, the outage capacity, and the ergodic capacity. The obtained analytical results are corroborated by an independent simulation method.

## 1. Introduction

It is well known that the number of devices and services in the field of wireless communications has been constantly growing, so the introduction of new concepts capable of meeting the set of demands with the resources available has become a necessity [1]. It is expected that 5G and beyond 5G technologies will provide a higher bandwidth, better coverage, reliable connections, energy savings, and an extremely low latency for various classes of users [2]. They also represent an essential component in the development of IoT (Internet of Things) systems, which assume the connection of a high number of devices, sensors, objects, and applications to the Internet. These applications will be able to collect huge amounts of data from various devices and sensors and can be encountered in a wide range of applications, from various commercial, health, and security applications to industrial applications, known as the Industrial Internet of Things [2]. The role of 5G and beyond 5G networks will be to provide high-speed Internet connections for the collection, transmission, control, and processing of data. Due to increasing challenges concerning the provision of 5G services and their applications in various environments, even the extension of conventional telecommunication systems to non-terrestrial ones has been proposed, including satellites and aerial systems [3]. As the integration of satellite–aerial–terrestrial networks enables improved support for diverse IoT applications, the spectrum and energy efficiencies of these systems have also recently attracted considerable attention [4,5,6].

As the concept of the IoT brings an ever-increasing number of low-power devices, it is obvious that the task of massive data transfer creates the need for additional spectrum resources, more efficient spectrum access, and innovative approaches [7]. According to the current allocation policy, the majority of the spectrum is already allocated, and the usage of novel techniques for efficient spectrum usage is obligatory [8]. Cognitive radio (CR) represents an innovative concept with the potential to solve the problem of spectrum scarcity [9] and has already been recognized in the literature as an important, promising approach for IoT applications [10]. Generally, two types of users can be distinguished in the concept of cognitive radio: primary and secondary users. While the primary users (PUs) are the licensed users of the spectrum, low utilization of the spectrum is prevented by allowing secondary users (SUs) to simultaneously use the resources under predefined conditions [11]. Depending on the spectrum-sharing policy used between two user classes, the following concepts can be distinguished: overlay, interweave, and underlay [12]. In the interweave concept of cognitive radio, secondary users are allowed to transmit when the channel is idle, based on the results of spectrum sensing. In the overlay concept, the secondary user is allowed to transmit under the assumption that it also helps the PU’s signal transmission and is based on a cooperation technique. The applications of interweave and overlay approaches in cognitive IoT systems were analyzed in [13,14].

In the underlay concept, the simultaneous communication of SUs with PUs is enabled, provided that harmful interference to the PUs is prevented by using the transmit power adaptation scheme [15]. If the perfect channel state information (CSI) is available, the level of interference occurring at the PU receiver can vary within certain tolerable limits. However, the realistic dynamic environment is characterized by constantly changing conditions, resulting in a practically erroneous or outdated CSI even when using concepts for which the necessary feedback in the system exists [16]. As IoT nodes are usually low-power and computationally limited, the approach based on statistical CSI can be beneficial for energy efficiency as it simplifies the extensive calculations needed for power adaptation in the conventional cognitive underlay concept. A performance analysis for cognitive systems with applied statistical CSI conditions was provided in [16], while a self-sustainable cognitive radio relaying system was analyzed in [17].

Another important issue related to IoT technology is the use of the large number of devices that the system relies on. Powering the devices is also a challenge. On one hand, even in scenarios in which the devices can be equipped with batteries, their lifespans are limited. On the other hand, due to the large number of energy-constrained wireless sensors and the potentially high-risk environment, battery replacement in the devices is not always straightforward. In order to ensure a longer lifetime of these sensors, the focus should be shifted to the use of wireless energy harvesting (EH) techniques, which represent a significant shift in green communications by enabling sensors in a network to harvest energy from various sources. A suitable energy source can be used for harvesting and accumulating energy in sensors, turning it into usable electrical energy. In this way, the lifetime of the batteries can be extended, or their usage can be replaced by a more practical approach. It is known that the EH process can be realized from various energy sources, such as solar, wind, vibration, and radio frequency (RF) signals [18]. 

An important advantage of using RF signals for EH arises from their capability to carry both energy and information; such an approach is used in the concept of simultaneous wireless information and power transmission (SWIPT) [19,20]. Using this approach, it is possible to enable the powering of sensors that do not have enough energy to transmit and process information. In SWIPT, there are two protocols used to transmit power information wirelessly: time switching (TS) and power splitting (PS) [21,22]. In addition to collecting energy from sources in the environment, energy can also be collected from a source intentionally dedicated to that purpose, called a power beacon (PB) [23,24]. The powering of a cooperative cognitive radio network by a dedicated power beacon was analyzed in [25], while an analysis of a multi-hop cognitive underlay network powered by a PB was presented in [26]. 

However, in the previously mentioned literature, cognitive radio systems using energy harvesting techniques were analyzed for scenarios in which a cognitive user demonstrated static behavior, which meant that the distances between the network nodes were represented by deterministic constants. This approach is not realistic in the generation of a novel system that is characterized by high user mobility, which significantly affects the performance of the wireless system due to the variability of the reception power [27]. Therefore, proper channel modeling relies on the predicting sensors’ movement models, which encompass different approaches [28]. In the literature, the two most represented models are the random direction (RD) and random waypoint (RWP) models. The first model uses a nonuniform spatial distribution [29], while the second one applies uniform spatial distribution [30]. RWP can describe the user’s movement through three different patterns, depending on whether the user is moving through 1D, 2D, or 3D systems [31,32].

### 1.1. Related Work

A performance analysis of a 5G cooperative network with static users and RF energy harvesting was presented in [33], while the performances of a static network whose source was powered by a PB were provided in [34]. The performance of a wirelessly powered network assisted with a dedicated power beacon using a TS protocol was analyzed in [35] for an interference-limited environment. The achievable capacity was calculated for different transmission schemes and power and rate adaptations for the case in which fading undergoes Rayleigh distribution. Another case of analyzing system performances in wirelessly powered communication network with a TS protocol was presented in [36] for the case of Rician fading distribution. The analysis of an SWIPT-based underlay cognitive radio network was presented in [37], while a scenario with multiple power beacons in a cognitive radio network was analyzed in [38].

As previously emphasized, wireless networks with static users were analyzed in most papers. This does not represent the usual behavior of users in the novel generation of wireless communication systems in which users are, in most cases, mobile, e.g., cellular users in cars, trains, and buses and pedestrians on streets. A performance analysis of a wireless system with a mobile receiver was provided for the Nakagami-*m* fading environment in [32], where the closed-form expressions for the system outage probability (OP) were derived for the applied RWP mobility model. The system performances of a wirelessly powered communication network were provided in [39,40] for the Nakagami-*m* fading environment, examining the case in which the source is powered by a designated power beacon and the mobility of the receiver node is described using the RWP model.

The impact of mobility on system performance was presented in [31] for a more complex η-μ fading distribution used to model the dynamic behavior of non-homogeneous fading, while the mobile receiver was described using the RWP mobility model. The analytical results for the ergodic channel capacity for generalized fading distribution were presented in [41], and the effects of mobility were evaluated in [42] for the k-μ generalized fading model (including Nakagami-*m* and Rician fading distribution as special cases); in both papers, the movement of the mobile receiver was described using the RWP model. Another mobile wireless network with Rician fading distribution was discussed in [43], and closed-form expressions for the outage probability in an interference-limited environment with a Rician fading channel were derived. In [44], the impact of mobility on a generalized α-µ fading distribution was analyzed. The authors of [28,31,41,45] analyzed the effects of mobility on the performances of communication systems by engaging the RWP model, while the influence of the RD mobility model was assessed in [46,47]. A comparison of these two models’ impact on performance was described in [27,48].

Additionally, [49] expanded the analysis of a system with a mobile receiver in a fading environment to the case in which the transmission of the cognitive node is in accordance with the imposed limitation of a primary network and an available, imperfect CSI [49]. It was assumed that the propagation environment is subject to Rayleigh fading distribution as a special case of Nakagami-*m* fading distribution. 

Our work is motivated by the fact that there is a growing number of connected IoT devices and unsolved issues concerning their supply, as well as the increasing need for efficient solutions for spectrum access. We analyze a system in which the transmitter is wirelessly powered using a PB, while efficient spectrum access and information transfer are enabled by simultaneous spectrum usage with the primary users without jeopardizing the conditions imposed by the primary network. The analysis encompasses a realistic scenario in which the receiver node is mobile, and the obtained performance represents the guidelines for the design of energy- and spectrum-constrained IoT systems with mobile receivers. In Table 1, a summary of related works is shown, demonstrating that there is no available research in the field of wirelessly powered cognitive radio networks with statistical CSI and random mobility, which we provide in this paper, filling the gap in the literature.

### 1.2. Contribution and Organization

In this paper we provide a performance analysis of an underlay cognitive radio system that is powered by a dedicated power beacon based on a time-switching protocol. The primary user constraints are defined by an interference threshold and a maximal primary user outage probability, which are satisfied under the assumption that statistical CSI is available to the secondary user of the spectrum. The contributions of this paper are as follows:

- We propose a cognitive radio system model powered by a dedicated power source in which the SU-Rx is mobile and the primary system is protected based on a tolerable interference level. We provide an analysis for the realistic scenario in which statistical CSI is available. Secondary user mobility is modeled using the widely accepted RWP model;
- We derive the analytical expressions for the probability density function of the received SNR and the outage probability of the secondary user;
- Based on the outage probability results, we calculate the outage capacity of the system, which is a relevant performance measure for applications with delay constraints;
- We derive the closed-form ergodic capacity expression relevant for applications with no delay requirements;
- The novel expressions derived in the paper are valid for the general case of the Nakagami-*m* fading environment, which encompasses Rayleigh and approximately Rician fading scenarios as special cases;
- Analytical results are confirmed using an independent simulation method. We analyze the impact of all system and channel parameters to the considered performance metrics.

In the following sections, we present a complete analysis in detail. In Section 2, the system and channel models are presented, in addition to the list of symbols shown in Table 2, which are used for further analysis. The outage performance analysis is provided in Section 3, and the ergodic capacity is analyzed in Section 4. The numerical results and discussion are provided in Section 5, and the conclusion is provided in Section 6. 

## 2. System and Channel Model

**Figure 1:**
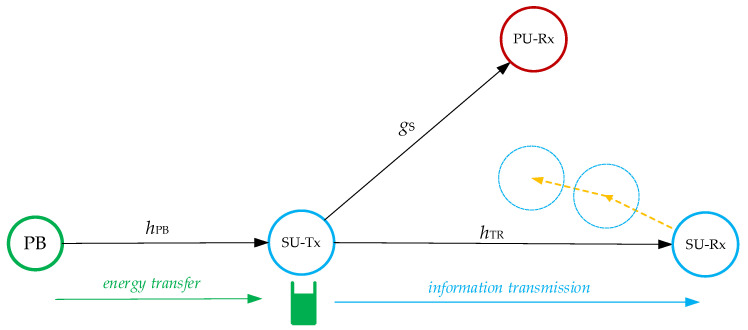
Model of the power-beacon-assisted underlay cognitive network with a random mobility user.

The wirelessly powered cognitive radio system is shown in Figure 1. The secondary network consists of the energy-constrained secondary transmitter (SU-Tx) and the secondary receiver (SU-Rx), which are allowed to exchange information as long as the interference produced at the primary user receiver is below a predetermined threshold. Further, we assume the SU-Tx does not have its own power supply but harvests energy from the dedicated power beacon. Energy harvesting and information transmission at the SU-Tx are based on the time-switching protocol. According to the applied protocol, all the energy harvested during a scheduled time frame is used for information transmission.

The secondary cognitive transmitter has a fixed position at a distance *D_PB_* from the power beacon node. The fading envelope in the channel from the PB to the SU-Tx *h_PB_* follows the Nakagami-*m* distribution, while the channel power gain $\gamma_{1}={\left|h_{PB}\right|}^{2}$ is distributed in accordance with Gamma distribution. Then, the probability density function (PDF) of a random variable $\gamma_{1}$ is provided by:

(1) $$f_{\gamma_{1}}\left(\gamma \right)=\frac{1}{\Gamma (m_{1})}{\left(\frac{m_{1}}{\Omega_{1}}\right)}^{m_{1}}\gamma^{m_{1}-1}\exp\left(-\frac{m_{1}}{\Omega_{1}}\gamma \right),$$

where $\Omega_{1}=E[{\left|h_{PB}\right|}^{2}]$ and *m*_1_ denotes the Nakagami-*m* fading parameter. It is well known that the Nakagami-*m* model encompasses a wide range of propagation scenarios, including the Rayleigh and Rice models as special cases. When the fading parameter is *m*_1_ = 1, Nakagami-*m* simplifies to the Rayleigh distribution, while in the case in which the propagation has a strong line-of-sight component [50], the parameter *m*_1_ has greater values. The power ratio *k* between the line-of-sight and the scattered components and the power of the scattered waves σ^2^ represent the Rician parameters, and their relation with the Nakagami-*m* parameters *m* and Ω can be expressed by the relations $m=\frac{1+k^{2}}{2k+1}$ and $\sigma^{2}=\frac{\Omega }{2}\left(1-\sqrt{1-m^{-1}}\right)$.

We analyze the realistic scenario in which the secondary receiver is not at a fixed location and is moving randomly. The movement of the secondary user receiver (SU-Rx) is modeled using the widely accepted RWP mobility model. Depending on the type of movement of the receiver, this model can be used for modeling paths in three different network topologies or dimensions.

As the SU-Rx is mobile, the distance between the fixed secondary transmitter and the receiver is represented by the random variable *r*. It is assumed that the mobile receiver is within a maximal distance *D* from the transmitter, i.e.,0≤r≤D, and the probability density function (PDF) of the distance *r* between the SU-Tx and SU-Rx is provided by [27]:

(2) $$f_{r}(r)=\sum_{l=1}^{n}\frac{B_{l}}{D^{\beta_{l}+1}}r^{\beta_{l}},$$

where the coefficients *B_l_* and *β_l_*, *l* = 1, …, *n*, which correspond to various topologies of the SU movement in one, two, or three dimensions, are provided in Table 3 [28].

The secondary network uses the spectrum dedicated to the primary user based on the underlay paradigm, which means that secondary users can perform transmissions concurrent with the primary users of the spectrum as long as the interference generated at the primary receiver does not exceed a permissible threshold. In this paper, we assume a realistic scenario in which only statistical CSI is available to the secondary network, i.e., only the mean channel power gain values (long-term CSI) are known. As the strict interference power constraint cannot be satisfied in this scenario, the PU’s outage probability constraint is applied. In this case, the peak interference threshold at the primary receiver can be exceeded, but only with a permitted maximum tolerable outage probability of the primary network. As the cognitive secondary network shares the spectrum with the primary user under the interference limit constraint, the maximal transmit power of the SU is determined by the maximal interference constraints imposed by the primary user of the spectrum.

**Figure 2:**
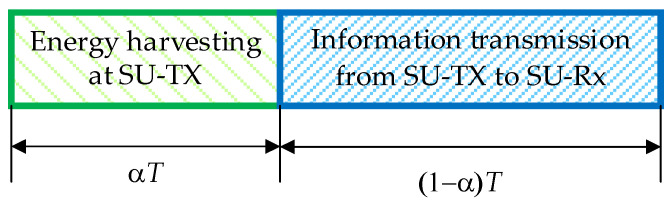
Transmission block structure in the TS scheme.

The transmission block structure is presented in Figure 2 for the case in which energy harvesting and information transmission are performed according to the TS protocol. Similar assumptions were used in [36] and [39]. Within a time frame interval of length *T*, the interval α*T*, α ∈ {0, 1} represents the fraction of the block time in which the SU-Tx harvests energy from the PB. The remaining block time, equal to (1 − α)*T*, is used for the transmission of information from the SU-Tx to the SU-Rx. 

The received signal at the SU-Tx node is provided by:

(3) $$y_{SU-Tx}=\sqrt{\frac{P_{PB}}{D_{PB}^{\delta }}}h_{PB}x_{PB}+n_{SU-Tx},$$

where *x_PB_* is the energy signal sent from the power beacon, *P_PB_* is the power of the signal from the power beacon, *D_PB_* is the distance from PB to SU-Tx, and *n_SU-Tx_* is an additive white Gaussian noise (AWGN) component. As can be seen from the previous equation, the path-loss effect is included in the analysis, and δ represents the path loss exponent.

Then, the energy harvested at the SU-Tx node can be expressed in the following form [17]:

(4) $$E_{H}=\eta \alpha T\frac{P_{PB}}{D_{PB}^{\delta }}{\left|h_{PB}\right|}^{2},$$

where *η* (0 < *η* < 1) is the energy conversion efficiency coefficient that depends on the characteristics of the energy harvester.

In accordance with the harvested energy and the remaining time used for signal transmission, the maximal available transmit power for the emitting of SU-Tx signal is equal to:

(5) $$P_{SU-Tx,EH}^{}=\frac{E_{H}}{\left(1-\alpha \right)T}=\frac{\eta \alpha P_{PB}}{(1-\alpha )D_{PB}^{\delta }}{\left|h_{PB}\right|}^{2}.$$

However, the transmit power of the cognitive SU-Tx should not exceed the maximal allowable value:

(6) $$P_{SU-Tx,\max}=k_{S}\frac{Q_{p}}{\Lambda_{S}},$$

which guarantees the fulfillment of the interference outage constraint. The coefficient *k_S_* adjusts the transmit power of the secondary user in accordance with the peak interference threshold *Q_p_* and the average value $\Lambda_{S}=E\left[{\left|g_{S}\right|}^{2}\right]$ of the channel power gain in the link from the SU-Tx to the PU-Rx ${\left|g_{S}\right|}^{2}$ such that the following maximal tolerable outage probability of primary network *P_out_*_,*PU*_ is not exceeded

(7) $$\mathrm{Pr}\left\{P_{SU-Tx}{\left|g_{S}\right|}^{2}<Q_{p}\right\}=1-P_{out,PU}.$$

Taking into account both constraints, the transmit power of the secondary user is provided as the minimum of the values *P_SU-Tx,EH_* and *P_SU-Tx_*_,max_, defined by Expressions (5) and (6), respectively. Therefore, it is defined by the following equation:

(8) $$P_{SU-Tx}^{}=\min\left(P_{SU-Tx,EH},P_{SU-Tx,\max}\right)=\left\{ \begin{array}{cc} \frac{\eta \alpha P_{PB}}{(1-\alpha )D_{PB}^{\delta }}{\left|h_{PB}\right|}^{2}, & \frac{\eta \alpha P_{PB}}{(1-\alpha )D_{PB}^{\delta }}{\left|h_{PB}\right|}^{2}\leq k_{S}\frac{Q_{p}}{\Lambda_{S}},\\ k_{S}\frac{Q_{p}}{\Lambda_{S}}, & \frac{\eta \alpha P_{PB}}{(1-\alpha )D_{PB}^{\delta }}{\left|h_{PB}\right|}^{2}>k_{S}\frac{Q_{p}}{\Lambda_{S}}. \end{array}\right.$$

The received baseband signal on SU-Rx is equal [33] to

(9) $$y_{SU-Rx}=\sqrt{\frac{P_{SU-Tx}}{r_{}^{\delta }}}h_{TR}x_{SU-Tx}+n_{SU-Rx},$$

where x*_SU-Tx_* is the information signal sent from the SU-Tx, *r* is the distance from the SU-Tx to the SU-Rx, and *n_SU-Rx_* is the AWGN component at the SU-Rx, with a mean power value $\sigma_{R}^{2}$. The channel gain coefficient from the SU-Tx to the SU-Rx is denoted by $h_{TR}$.

The instantaneous signal-to-noise ratio (SNR) γ at the SU-Rx is provided by the following equation:

(10) $$\gamma =\frac{P_{SU-Tx}{\left|h_{TR}\right|}^{2}}{r^{\delta }\sigma_{R}^{2}}=\left\{ \begin{array}{cc} K_{1}{\left|h_{PB}\right|}^{2}\frac{{\left|h_{TR}\right|}^{2}}{r^{\delta }}, & {\left|h_{PB}\right|}^{2}\leq Q,\\ K_{2}\frac{{\left|h_{TR}\right|}^{2}}{r^{\delta }}, & {\left|h_{PB}\right|}^{2}>Q, \end{array}\right.$$

where $K_{1}=\frac{\eta \alpha P_{PB}}{\sigma_{R}^{2}(1-\alpha )D_{PB}^{\delta }}$, $K_{2}=k_{S}\frac{Q_{p}}{N_{0}B\Lambda_{S}}$, and $Q=k_{S}\frac{Q_{p}}{\Lambda_{S}K_{1}}$.

Further, we also assume that the fading envelope in the link between the SU-Tx and the SU-Rx follows Nakagami-*m* distribution. Taking into account the movement of the SU-Rx and the variable distance *r* between the SU-Tx and SU-Rx, described by the Equation (2), the final expression for the PDF of the instantaneous channel power gain $\gamma_{2}$ is obtained via ([32], (7)):

(11) fγ2(x)=1Γ(m2)(m2Ω2)m2xm2−1∑i=1nBim2δ+βi+1 F11(m2+βi+1δ,1+m2+βi+1δ,−m2xΩ2),

where $\gamma_{2}={\left|h_{TR}\right|}^{2}r^{-\delta }$ and $\Omega_{2}=E[{\left|h_{TR}\right|}^{2}D^{-\delta }]$.

Then, instantaneous SNR expression can be written in the following form:

(12) $$\gamma =\left\{ \begin{array}{cc} \gamma_{K_{1}}\gamma_{1}, & \gamma_{1}\leq Q,\\ \gamma_{K_{2}}, & \gamma_{1}>Q, \end{array}\right.$$

where $\gamma_{K_{1}}=K_{1}\gamma_{2}$ and $\gamma_{K_{2}}=K_{2}\gamma_{2}$. The corresponding probability density functions (PDFs) of the random variables $\gamma_{K_{i}}$ and *i* = 1, 2 are provided by $f_{\gamma_{K_{i}}}\left(x\right)=\frac{1}{K_{i}}f_{\gamma_{i}}\left(\frac{x}{K_{i}}\right)$ ([51], (5–7)) and have the form:

(13) fγKi(x)=1Kim21Γ(m2)(m2Ω2)m2xm2−1∑i=1nBim2δ+βi+1 ×F11(m2+βi+1δ,1+m2+βi+1δ,−m2xKiΩ2).

## 3. Outage Capacity

In the following section, we present the analysis of the outage performances of the wirelessly powered cognitive radio system presented herein. First, we calculate the PDF of the random variable γ, provided by

(14) $$f_{\gamma }\left(u\right)=\int_{0}^{Q}f_{u,w}\left(u,w\right)dw+\int_{Q}^{\infty }f_{\gamma_{K2},\gamma_{1}}\left(u,w\right)dw,$$

where $f_{u,w}\left(u,w\right)$ is the joint PDF of the variables $u=\gamma_{K_{1}}\gamma_{1}$ and $w=\gamma_{1}$, and $f_{\gamma_{K2},\gamma_{1}}\left(u,w\right)$ is the joint PDF of variables $\gamma_{K_{2}}$ and $\gamma_{1}$.

The joint PDF of variables $u=\gamma_{K_{1}}\gamma_{1}$ and $w=\gamma_{1}$ can be obtained using the Jacobian transformation $f_{u,w}\left(u,w\right)=\left|J\right|f_{\gamma_{K_{1}}}\left(\frac{u}{w}\right)f_{\gamma_{1}}\left(w\right)$, where |J|=1/|w|. On the other hand, a joint PDF of the independent variables $\gamma_{K_{2}}$ and $\gamma_{1}$ can be obtained as $f_{\gamma_{K_{2}},\gamma_{1}}\left(u,w\right)=f_{\gamma_{K_{2}}}\left(u\right)\cdot f_{\gamma_{1}}\left(w\right)$. The PDF of instantaneous SNR from (14) is:

(15) $$f_{\gamma }\left(u\right)=\int_{0}^{Q}\left|J\right|f_{\gamma_{K_{1}}}\left(\frac{u}{w}\right)f_{\gamma_{1}}\left(w\right)dw+\int_{Q}^{\infty }f_{\gamma_{K_{2}}}\left(u\right)\cdot f_{\gamma_{1}}\left(w\right)dw.$$

Further, by replacing the PDF expressions (1) and (13) in (15), the following expression is obtained:

(16) fγ(u)=1Γ(m1)Γ(m2)(m1Ω1)m1(m2Ω2)m2um2−1K1m2∑i=1nBim2δ+βi+1 I1+1Γ(m1)Γ(m2)(m1Ω1)m1(m2Ω2)m2um2−1K2m2∑i=1nBim2δ+βi+1×F11(m2+βi+1δ,1+m2+βi+1δ,−m2uK2Ω2)I2,

where corresponding integrals are

(17) I1=∫0QF11(m2+βi+1δ,1+m2+βi+1δ,−m2uK1Ω2w)wm1−m2−1exp(−m1Ω1w)dw,

and

(18) $$I_{2}=\int_{Q}^{\infty }\;w^{m_{1}-1}\exp\left(-\frac{m_{1}}{\Omega_{1}}w\right)dw.$$

The mathematical transformations provided in Appendix A lead to a solution for the integrals *I*_1_ and *I*_2_ and the following final PDF expression for the instantaneous SNR at the SU-Rx:

(19) fγ(u)=Cum2−1K1m2∑i=1n∑k=0∞1k!(−m1Ω1)kBi(m2+βi+1δ)m2δ+βi+1 ×Qm1−m2+kG3,21,2(K1Ω2Qm2u|1−m1+m2−k,1,1+m2+βi+1δm2+βi+1δ,m2−m1−k)+Cum2−1K2m2(Ω1m1)m1Γ(m1,m1Ω1Q)∑i=1nBim2δ+βi+1×F11(m2+βi+1δ,1+m2+βi+1δ,−m2uK2Ω2),

where $C=\frac{1}{\Gamma (m_{1})}\frac{1}{\Gamma (m_{2})}{\left(\frac{m_{1}}{\Omega_{1}}\right)}^{m_{1}}{\left(\frac{m_{2}}{\Omega_{2}}\right)}^{m_{2}}$.

Further, the CDF expression for the random variable γ can be obtained via

(20) $$F_{\gamma }\left(\gamma_{th}\right)=\int_{0}^{\gamma_{th}}f_{\gamma }\left(u\right)du.$$

By replacing (19) in the previous equation, the CDF can be expressed as

(21) $$\begin{array}{c} F_{\gamma }\left(\gamma_{th}\right)=C\frac{1}{K_{1}^{m_{2}}}\sum_{i=1}^{n}\sum_{k=0}^{\infty }\frac{1}{k!}{\left(-\frac{m_{1}}{\Omega_{1}}\right)}^{k}B_{i}\frac{\left(m_{2}+\frac{\beta_{i}+1}{\delta }\right)}{m_{2}\delta +\beta_{i}+1}\;Q^{m_{1}-m_{2}+k}\;I_{3}\\ +C\frac{1}{K_{2}^{m_{2}}}{\left(\frac{\Omega_{1}}{m_{1}}\right)}^{m_{1}}\Gamma \left(m_{1},\frac{m_{1}}{\Omega_{1}}Q\right)\sum_{i=1}^{n}\frac{B_{i}}{m_{2}\delta +\beta_{i}+1}\;I_{4}, \end{array}$$

where

(22) $$I_{3}=\int_{0}^{\gamma_{th}}u^{m_{2}-1}G_{3,2}^{1,2}\left(\frac{K_{1}\Omega_{2}Q}{m_{2}u}|\begin{array}{c} \begin{array}{ccc} 1-m_{1}+m_{2}-k, & 1, & 1+m_{2}+\frac{\beta_{i}+1}{\delta } \end{array}\\ \begin{array}{cc} m_{2}+\frac{\beta_{i}+1}{\delta }, & m_{2}-m_{1}-k \end{array} \end{array}\right)du,$$

and

(23) I4=∫0γthum2−1F11(m2+βi+1δ,1+m2+βi+1δ,−m2uK2Ω2)du.

The integrals *I*_3_ and *I*_4_ can be solved in the exact closed form by applying the transformations described in Appendix B. Finally, the expression for the outage probability of the secondary link can be written in the following form:

(24) $$\begin{array}{ll} F_{\gamma }\left(\gamma_{th}\right) & =C\frac{\gamma_{th}^{m_{2}}}{K_{1}^{m_{2}}}\sum_{i=1}^{n}\sum_{k=0}^{\infty }\frac{1}{k!}{\left(-\frac{m_{1}}{\Omega_{1}}\right)}^{k}B_{i}\frac{\left(m_{2}+\frac{\beta_{i}+1}{\delta }\right)}{m_{2}\delta +\beta_{i}+1}\;Q^{m_{1}-m_{2}+k}\\ & \times G_{3,4}^{2,2}\left(\frac{m_{2}\gamma_{th}}{K_{1}\Omega_{2}Q}|\begin{array}{c} \begin{array}{ccc} 1-m_{2}, & 1-m_{2}-\frac{\beta_{i}+1}{\delta }, & 1-m_{2}+m_{1}+k \end{array}\\ \begin{array}{cccc} m_{1}-m_{2}+k, & 0, & -m_{2}-\frac{\beta_{i}+1}{\delta }, & -m_{2} \end{array} \end{array}\right)\\ & +C\frac{\gamma_{th}^{m_{2}}}{K_{2}^{m_{2}}}{\left(\frac{\Omega_{1}}{m_{1}}\right)}^{m_{1}}\Gamma \left(m_{1},\frac{m_{1}}{\Omega_{1}}Q\right)\sum_{i=1}^{n}B_{i}\frac{\left(m_{2}+\frac{\beta_{i}+1}{\delta }\right)}{m_{2}\delta +\beta_{i}+1}\;\\ & \times G_{2,3}^{1,2}\left(\frac{m_{2}\gamma_{th}}{K_{2}\Omega_{2}}|\begin{array}{c} \begin{array}{cc} 1-m_{2}, & 1-m_{2}-\frac{\beta_{i}+1}{\delta } \end{array}\\ \begin{array}{ccc} 0, & -m_{2}-\frac{\beta_{i}+1}{\delta }, & -m_{2} \end{array} \end{array}\right). \end{array}$$

Another important feature describing the secondary system is the achievable throughput. To obtain the achievable throughput, the outage capacity is calculated using the following equation [52]:

(25) $$C_{OUT}=(1-P_{OUT}(\gamma_{th}))log_{2}(1+\gamma_{th}),$$

where $P_{OUT}\left(\gamma_{th}\right)=F_{\gamma }\left(\gamma_{th}\right)$.

The outage capacity is defined as the maximum data rate that can be achieved. Further, the achievable throughput is expressed by [35]:

(26) $$T_{OUT}^{}=(1-\alpha )C_{OUT}.$$

## 4. Ergodic Capacity

In this section, we analyze the ergodic capacity of the wirelessly powered secondary link, which is one of the most important performance metrics appropriate for applications with no delay requirements. The ergodic capacity represents a maximal long-term rate that can be achieved over a channel with an arbitrary small probability of error [52], and it can be calculated via

(27) $$C_{erg}=\frac{1}{\log_{e}2}\int_{0}^{\infty }\log_{e}\left(1+u\right)f_{\gamma }\left(u\right)du.$$

Knowing the ergodic capacity, the achievable ergodic throughput can be obtained via

(28) $$T_{erg}^{}=(1-\alpha )C_{erg}.$$

By replacing the PDF represented by Meijer functions using the procedure provided in Appendix C and the integration provided in (27), the expression for the ergodic capacity of a wirelessly powered secondary link is:

(29) $$\begin{array}{ll} C_{erg} & =\frac{1}{\log_{e}2}\frac{C}{K_{1}^{m_{2}}}\sum_{i=1}^{n}\sum_{k=0}^{\infty }\frac{1}{k!}{\left(-\frac{m_{1}}{\Omega_{1}}\right)}^{k}B_{i}\frac{\left(m_{2}+\frac{\beta_{i}+1}{\delta }\right)}{m_{2}\delta +\beta_{i}+1}Q^{m_{1}-m_{2}+k}\\ & \times G_{4,5}^{4,2}\left(\frac{m_{2}}{K_{1}\Omega_{2}Q}|\begin{array}{c} \begin{array}{cc} \begin{array}{cc} \begin{array}{cc} 1-m_{2}-\frac{\beta_{i}+1}{\delta }, & -m_{2}, \end{array} & 1-m_{2}, \end{array} & 1-m_{2}+m_{1}+k \end{array}\\ \begin{array}{cc} \begin{array}{ccc} \begin{array}{cc} m_{1}-m_{2}+k, & 0, \end{array} & -m_{2}, & -m_{2}, \end{array} & -m_{2}-\frac{\beta_{i}+1}{\delta } \end{array} \end{array}\right)\\ & +\frac{1}{\log_{e}2}\frac{C}{K_{2}^{m_{2}}}{\left(\frac{\Omega_{1}}{m_{1}}\right)}^{m_{1}}\Gamma \left(m_{1},\frac{m_{1}}{\Omega_{1}}Q\right)\sum_{i=1}^{n}B_{i}\frac{\left(m_{2}+\frac{\beta_{i}+1}{\delta }\right)}{m_{2}\delta +\beta_{i}+1}\;\\ & \times G_{3,4}^{3,2}\left(\frac{m_{2}}{K_{2}\Omega_{2}}|\begin{array}{c} \begin{array}{ccc} 1-m_{2}-\frac{\beta_{i}+1}{\delta }, & -m_{2}, & 1-m_{2} \end{array}\\ \begin{array}{cccc} 0, & -m_{2}, & -m_{2}, & -m_{2}-\frac{\beta_{i}+1}{\delta } \end{array} \end{array}\right). \end{array}$$

## 5. Numerical Results

**Figure 3:**
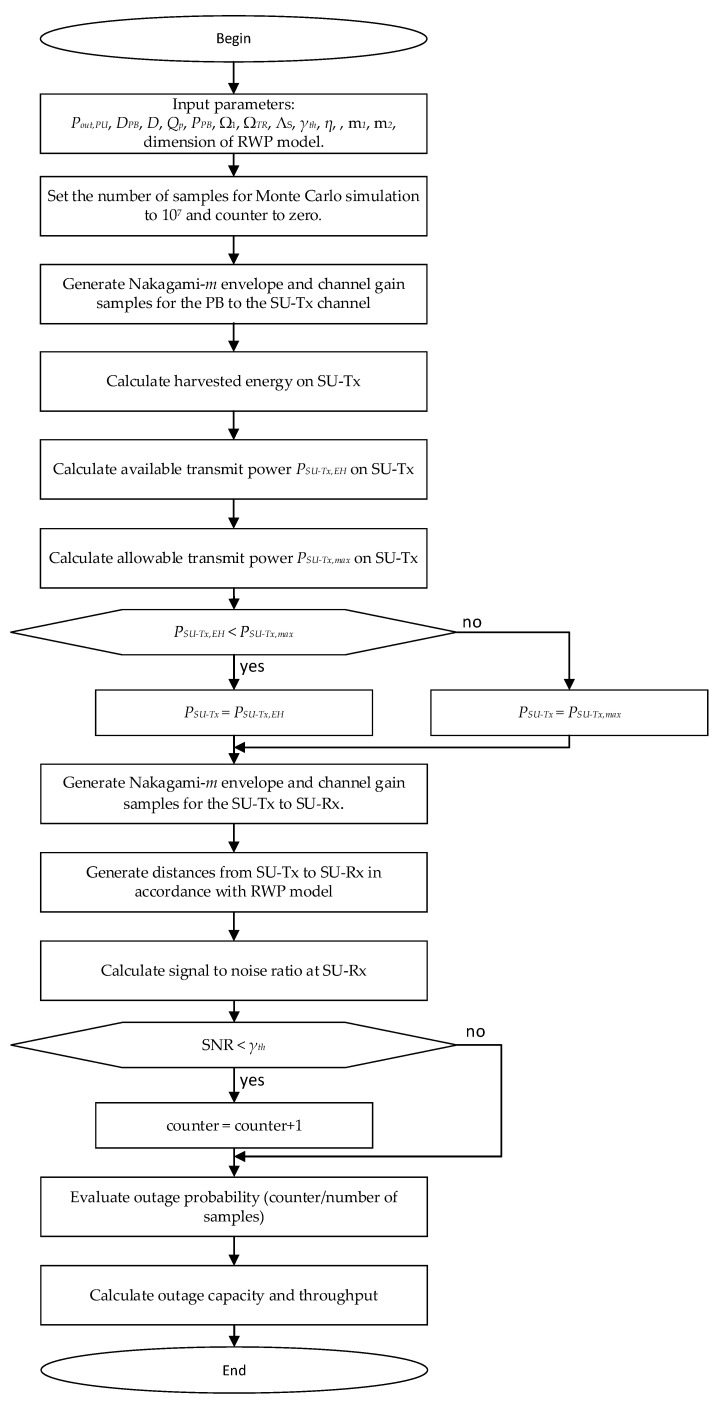
Flowchart of the steps used for the performance evaluation.

In this section, we present the numerical results of the system performances analyzed in previous chapters. All numerical results were confirmed by an independent Monte Carlo simulation method [53] implemented in MATLAB, based on generated waveform sequences with *L* = 10^7^ samples in accordance with [50,54]. The flowchart presented in Figure 3 shows the steps used in the system performance evaluation. Analytical expressions are represented by Equations (24) and (29). Various parameters that the system’s performance depended on were changed, and an analysis of the obtained results was carried out. 

**Figure 4:**
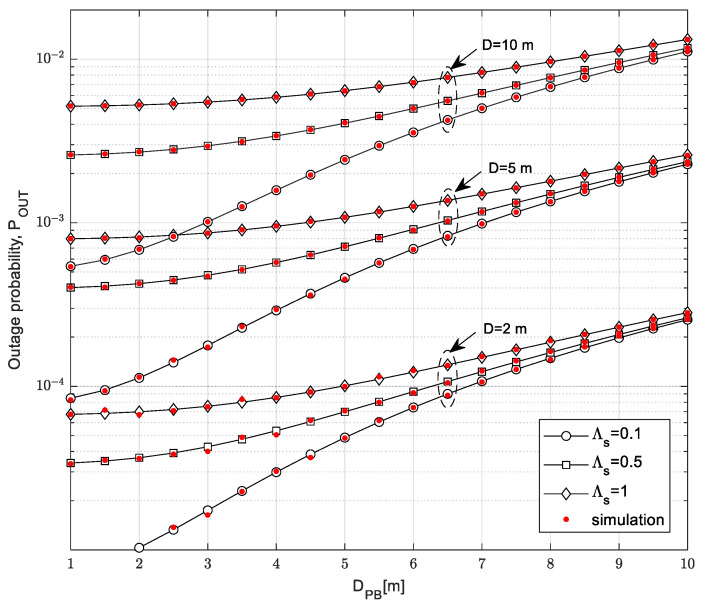
Outage probability vs. distance from the power beacon to the secondary transmitter *D*_PB_.

**Figure 5:**
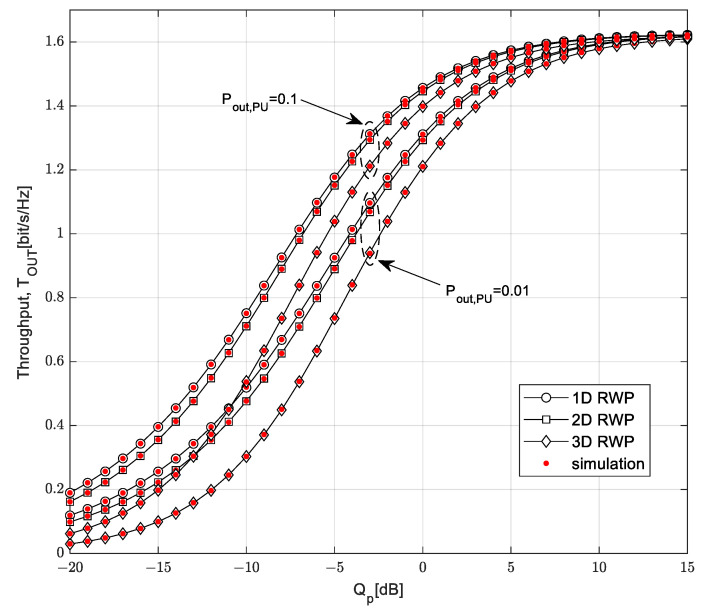
Throughput *T*_OUT_ vs. interference threshold *Q_p_* for various mobility models.

**Figure 6:**
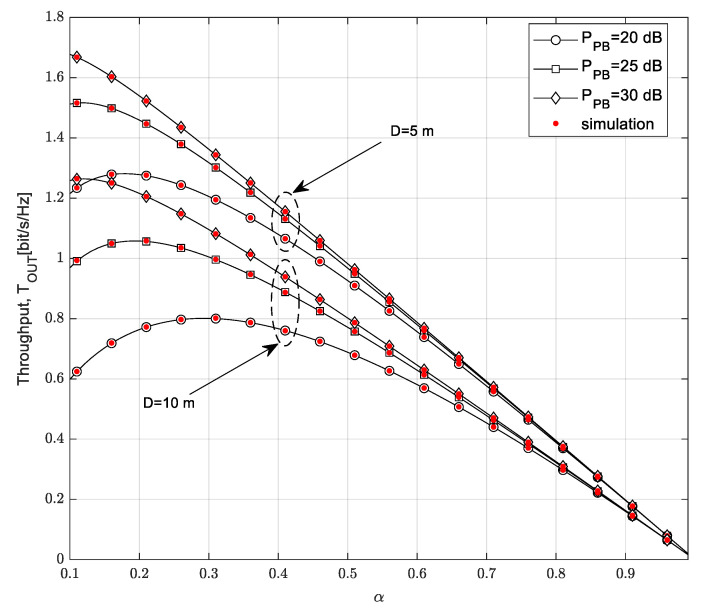
Throughput *T_OUT_* vs. time-switching factor *α*.

**Figure 7:**
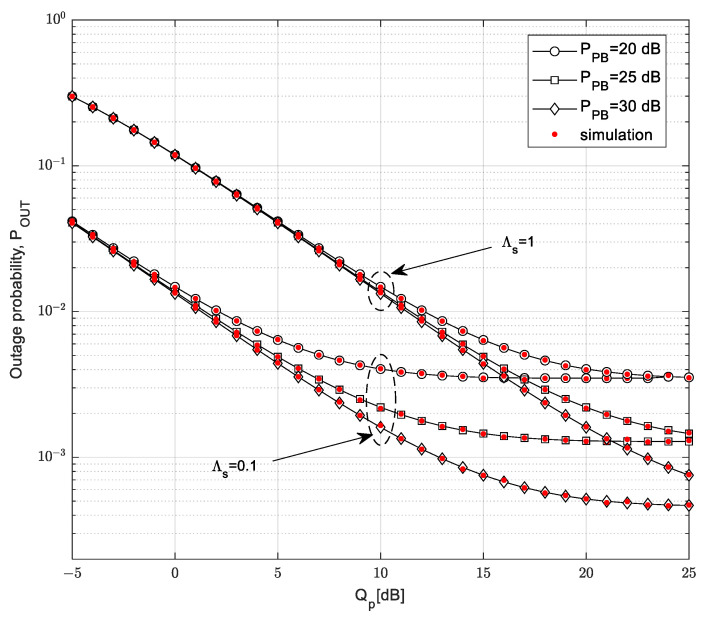
Outage probability vs. interference threshold *Q_p_* for various *P_PB_* values.

**Figure 8:**
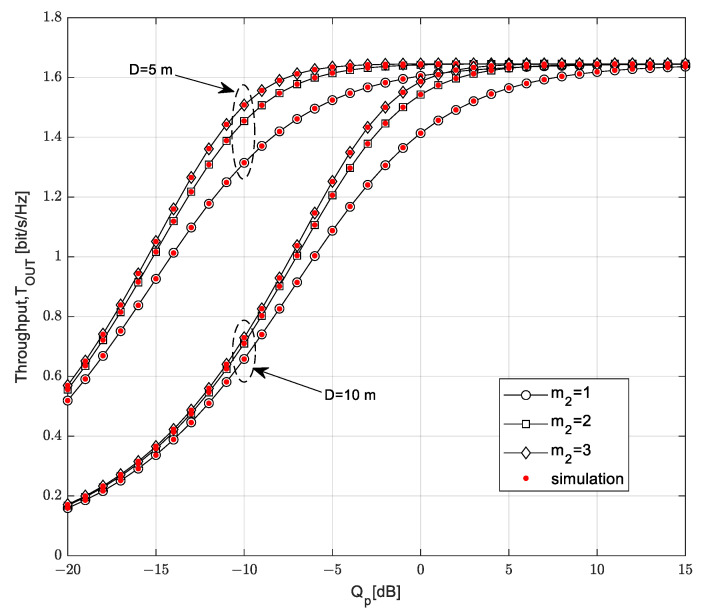
Throughput *T*_OUT_ vs. interference threshold *Q_p_* for various distances *D*.

**Figure 9:**
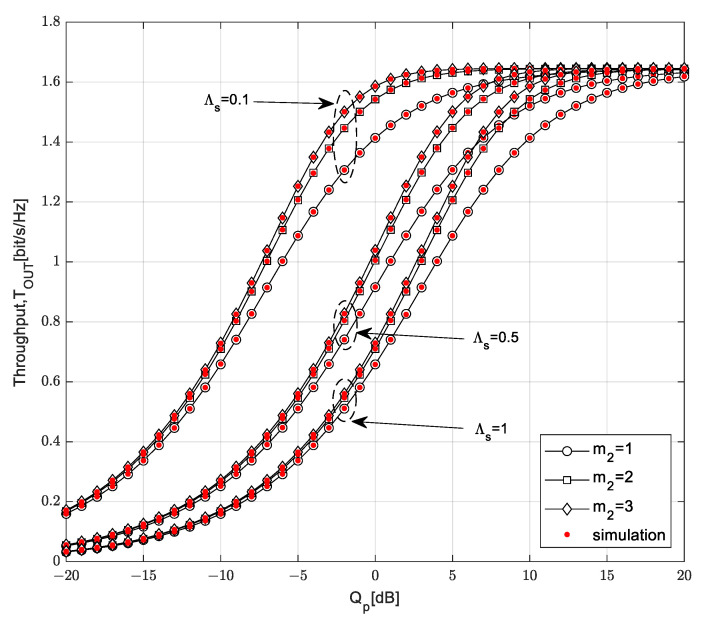
Throughput *T*_OUT_ vs. interference threshold *Q_p_* for various channel power gains Λ_S_.

**Figure 10:**
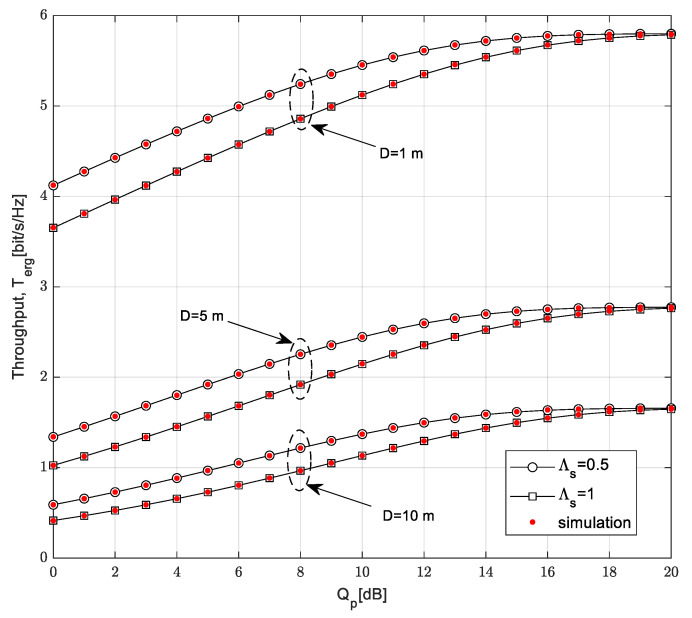
Throughput *T*_erg_ vs. interference threshold *Q_p_* for various distances *D*.

**Figure 11:**
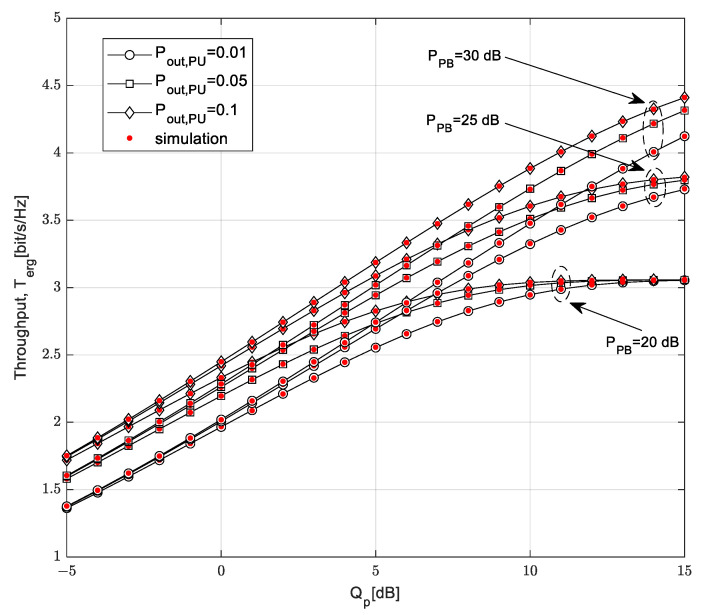
Ergodic throughput vs. interference threshold *Q_p_* for various *P_PB_* values.

**Figure 12:**
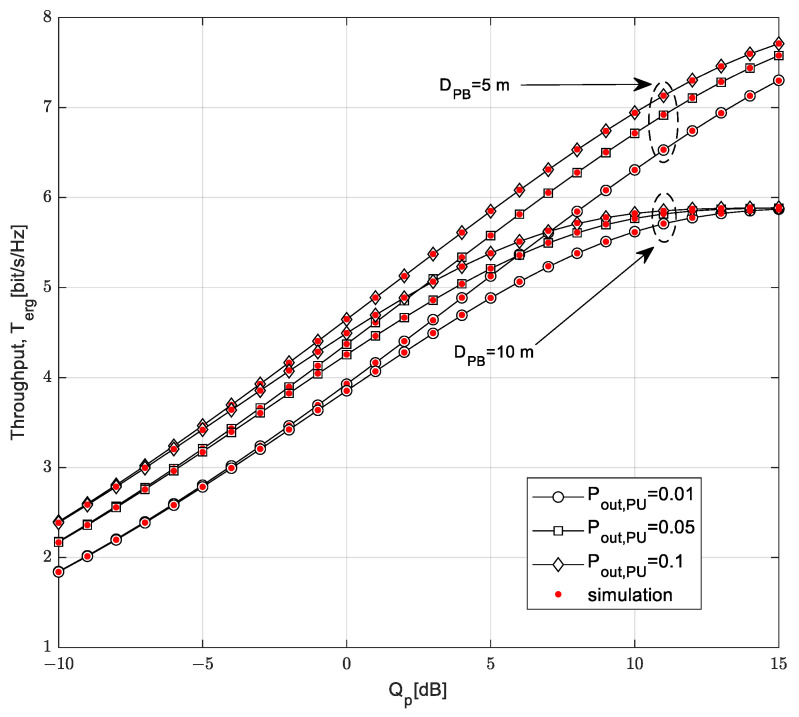
Ergodic throughput vs. interference threshold *Q_p_* for different probability values *P_out, PU_*.

**Figure 13:**
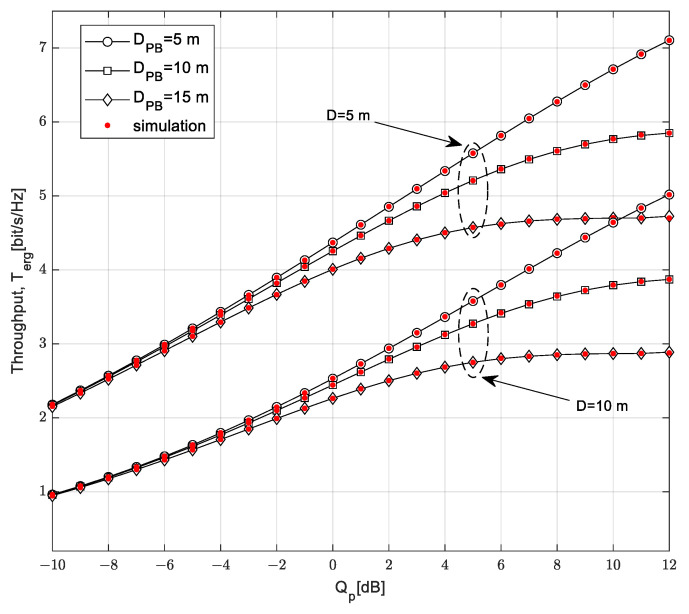
Ergodic throughput vs. interference threshold *Q_p_* for various distances *D_PB_* and *D*.

**Figure 14:**
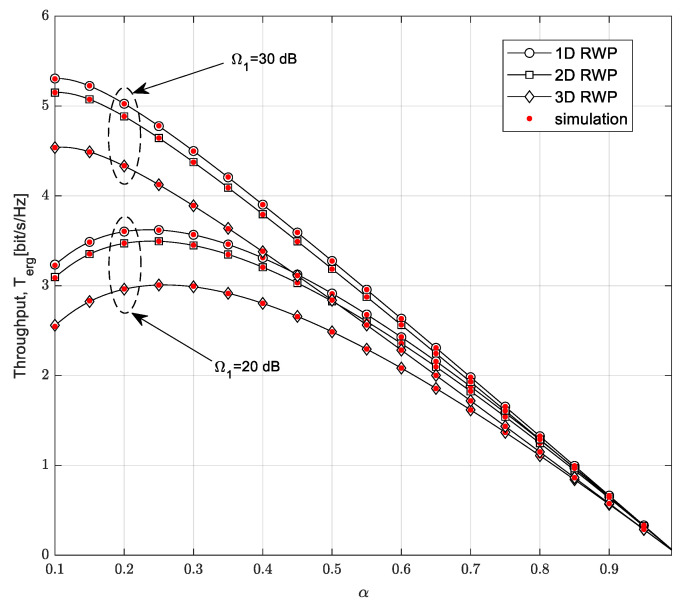
Ergodic throughput vs. time-switching factor *α* for different mobility models.

For the proposed system, the following important secondary system performance metrics were considered: the outage probability, the throughput based on outage capacity, and the throughput based on ergodic capacity. The simulation parameters used for all considered scenarios presented in Figure 4, Figure 5, Figure 6, Figure 7, Figure 8, Figure 9, Figure 10, Figure 11, Figure 12, Figure 13 and Figure 14 are provided in Table 4. The system and channel parameters presented in Table 4 have significant influence on system performance, and they were varied in the presented analysis in order to provide important conclusions considering the system design.

Figure 4 shows the dependence of the outage probability of the distance *D_PB_* from the PB to the SU-Tx. The curves on the graph are presented for different values of the maximum distance from the SU-Tx to the SU-Rx and the mean channel power gain Λ_S_ in the channel from the SU-Tx to the PU-Rx for the outage threshold equal *γ_th_* = −5 dB. The time-switching factor was *α* = 0.5, which means that equal time intervals were dedicated for the wireless power transfer and the information transfer in the secondary system. In the considered scenario, the 1D path model was assumed, meaning that the SU-Rx moves according to the RWP model along a straight line. The limitations imposed by the primary networks were: the interference threshold *Q_p_* = 15 dB and the outage probability of the primary network *P*_out,PU_ = 0.01. 

From the obtained results, it can be noted that the increase in the distance between the PB and the SU-Tx also increases the outage probability values. The reason for this effect is the fact that the increase in the distance from the PB to the SU-Tx decreases the received signal power at the SU-Tx and the harvested energy on the SU-Tx, leading to its lower transmission power. In addition, greater values of the maximum distance from the SU-Tx to the SU-Rx have a degrading impact on the outage probability performances as the received power at the SU-Rx is smaller and the SNR at the SU-Rx is lower. The propagation characteristics in the channel from the SU-Tx to the PU-Rx also significantly impact outage performances. With a decrease in the mean channel power gain value in the channel from the SU-Tx to the PU-Rx, the outage probability of the SU system decreases because the limitation of the transmitted power at the SU-Tx is weaker. By increasing the distance from the PB to the SU-Tx, the value of the mean channel power gain in the channel from the secondary transmitter to the primary receiver has a less significant impact on the outage probability. In this case, a smaller amount of energy is harvested, resulting in a smaller transmitted power of the SU when the interference limitation of the transmitted power does not have a dominant impact. On the other hand, small values of the distance *D_PB_* result in high amounts of harvested energy when the interference constraint imposed by the PU is dominant. Therefore, in this case, when the channel power gain is high, variations in the values of *D_PB_* do not dominantly influence outage performances, and they have similar values in the range of up to 3 m.

The dependence of the throughput *T_OUT_* on the interference threshold *Q_p_* is analyzed in Figure 5 for two values of the permitted primary network outage probability, *P*_out,PU_, equal to 0.1 and 0.01, respectively. In this figure, we also consider scenarios with different SU-Rx mobility models. 

In accordance with the expectations, better system performance was achieved when the permitted outage probability of the PU network and the required interference threshold had greater values. With a more lenient condition for the harmful interference on the primary receiver, which was expressed through higher values of the interference threshold and the outage probability of the primary network, a higher value of transmission power was allowed on the SU-Tx. For interference threshold values higher than 10 dB, the throughput values entered the saturation range, and they were not dominantly dependent on the outage probability of the primary network. In this scenario, the transmission power of the SU solely depended on the harvested energy and not on limitations caused by harmful interference on the primary user. Finally, Figure 5 shows the throughput for different mobility models, demonstrating performances in the cases of SU-Rx movement along the line path, circle surface, or inside the sphere. It can be noted that the best results are obtained when movement of SU-Rx can be described using the 1D model path.

The dependence of throughput on the time-switching factor α is demonstrated in Figure 6. Additionally, the impact of the transmission power of the PB and the maximum distance from the SU-Tx to the SU-Rx are analyzed for the case in which the SU-Rx moves along 1D path model.

The throughput of the secondary network increases by increasing the time-switching factor α up to a certain value; after this value, it begins to decrease as although a higher α value results in a higher amount of harvested energy at the SU-Tx, the dominant impact has a smaller amount of time dedicated to the transmission of information. For the same value of time-switching factor α, higher throughput values are achieved if the transmission power of the PB is higher because more energy is harvested at the SU-Tx in this case, resulting in a higher transmit power of the SU. The influence of the PB’s power values is greater in the case in which *D* = 10 m than in the case in which *D* = 5 m. In addition, the smaller value of the maximum distance from the SU-Tx to the SU-Rx leads to greater throughput values as the receiver power at the SU-Rx is higher, resulting in a higher received SNR at the SU-Rx.

The dependence of outage probability on the interference threshold *Q_p_* for different values of the transmission power of the PB and different values of the mean channel power gain from the SU-Tx to the PU-Rx is shown in Figure 7, demonstrating the case in which the primary network outage probability is *P*_out,PU_ = 0.1 and the outage threshold is *γ_th_* = −5 dB, while the secondary receiver moves over the 2D area with a maximum distance from the SU-Tx of *D* = 5 m. 

The outage probability of the SU decreases with the increase in the interference threshold *Q_p_*, as the SU-Tx can transmit with higher permitted power. With high values of the interference threshold *Q_p_*, the outage probability enters the saturation region. For smaller values of the PB transmit power, the dependence enters the saturation region for a smaller *Q_p_*, while with higher values of the PB power, the saturation occurs for a higher interference threshold *Q_p_*. For high values of the interference threshold, the transmit power of the SU-Tx depends completely on the harvested energy, so in this scenario only the PB power has an influence on the collected energy. Furthermore, more energy results in a higher transmit power of the SU-Tx, which leads to a smaller outage probability. For small values of the interference threshold *Q_p_*, the outage probability is not dependent on the power of the PB because the limitation caused by harmful interference on the PU-RX has the dominant impact. It can be noted that better system performances are obtained for lower values of mean channel power gain in the channel from the SU-Tx to the PU-Rx, which is the effect also shown in Figure 4. Additionally, for a higher interference threshold and the same power value of the PB, the outage probability in the saturation region becomes independent of the mean channel power gain in the channel from the SU-Tx to the PU-Rx because the transmission power of the SU-Tx depends only on the harvested energy from the PB.

The throughput *T*_OUT_ is presented in Figure 8 as a function of the interference threshold *Q_p_*. The influence of the maximum distance between the SU-Tx and the SU-Rx is analyzed, as is the impact of the Nakagami-*m* fading parameter *m*_2_ in the SU channel (from the SU-Tx to the SU-Rx). 

The throughput *T*_OUT_ of the secondary network increases with the increase in the interference threshold, while saturation occurs for larger values of the interference threshold. In the region of a smaller *Q_p_* with no saturation effect, the throughput depends on all analyzed parameters. In accordance with the expectations, a smaller throughput is achieved if the maximum distance from the SU-Tx to the SU-Rx is higher: in that case, the mean value of the received power at the SU-Rx is smaller. A higher value of the Nakagami-*m* fading parameter *m*_2_ in the channel from the SU-Tx to the SU-Rx leads to a higher value of the achievable throughput. It can be noted that the throughput increases with the increase in the interference threshold to a certain limit, when the saturation region begins. In the case in which the maximum distance from the SU-Tx to the SU-Rx is *D* = 5 m, the saturation occurs at a lower *Q_p_*, and a further increase in the interference threshold does not change the throughput values. At high values of the interference threshold, there is no significant difference between the throughput values for the maximum distance *D* = 5 m and *D* = 10 m and for the analyzed values of the Nakagami-*m* fading parameter *m*_2_ in the SU link. By increasing the interference threshold, the limitation conditioned by the harmful interference on the primary user becomes weaker, so the secondary transmitter can use all the harvested energy for transmission.

The dependence of the throughput *T*_OUT_ on the interference threshold *Q_p_* is shown in Figure 9. In this scenario, the values of the mean channel power gain in the channel from the SU-Tx to the PU-Rx, as well as the Nakagami-*m* fading parameter *m*_2_ in the SU link, are varied. The mean channel power gain in Nakagami-*m* channel from the PB to the SU-Tx is Ω_1_ = 10 dB, the distance from the PB to the SU-Tx is *D_PB_* = 1 m, and the transmission power of the PB is *P_PB_* = 30 dB. It is assumed that the SU-Rx movement can be described with a 1D model. The maximum distance from the SU-Tx to the SU-Rx is *D* = 5 m, the outage probability of the primary network is *P*_out,PU_ = 0.01, the time-switching factor is *α* = 0.2, and the outage threshold is *γ_th_* = 5 dB.

For higher values of the interference threshold *Q_p_*, the throughput reaches the saturation value, the interference threshold value at which the saturation region begins depends on the mean channel power gain value of the channel from the SU-Tx to the PU-Rx. Both factors, the interference threshold *Q_p_* and the mean channel power gain in the channel from the SU-Tx to the PU-Rx, have influences on the limiting values conditioned by harmful interference on the primary user. A greater value of the Nakagami-*m* fading parameter *m*_2_ in the secondary user channel leads to a higher achievable throughput. Observing the behavior of curves for very small values of the interference threshold *Q_p_*, it can be concluded that for the same parameter of the mean channel power gain, Λ*_S_*, approximately the same values of throughput are achieved for different values of the fading parameter *m*_2_, as the allowed transmitted power from the secondary transmitter is very small in all cases.

The curves in Figure 10 show the dependence of the ergodic throughput on the interference threshold *Q_p_* for different values of the maximum distance from SU-Tx to SU-Rx and the mean channel power gain in the channel from the SU-Tx to the PU-Rx. The time-switching factor is *α* = 0.5, the mean channel power gain in the Nakagami-*m* channel from the PB to the SU-Tx is Ω_1_ = 10 dB, the distance from the PB to the SU-Tx is *D_PB_* = 5 m, and the transmission power of the PB is *P_PB_* = 20 dB. It is assumed that the SU-Rx moves along a 2D path model, while the mean channel power gain in the Nakagami-*m* channel from the SU-Tx to the SU-Rx is Ω*_TR_* = 20 dB, and the outage probability of the primary network is *P*_out,PU_ = 0.05.

By increasing the interference threshold, the ergodic throughput of the secondary network increases for an interference threshold smaller than 15 dB, while for values greater than 15 dB, the saturation effect occurs. A higher value of the interference threshold *Q_p_* leads to a greater value of the ergodic throughput because the SU-Tx is allowed to transmit with more power, so the received SNR at SU-Rx is higher. For values greater than 15 dB, the ergodic throughput depends solely on the harvested energy. By further increasing the interference threshold, the ergodic throughput becomes independent of the mean channel power gain in the channel from the SU-Tx to the PU-Rx. A smaller ergodic throughput is achieved if the maximum distance from the SU-Tx to the SU-Rx is higher because in that case, the received power at the SU-Rx is smaller. If the mean channel power gain in channel from the SU-Tx to the PU-Rx is higher, a smaller ergodic throughput is achieved because of the interference limitation at the primary user, leading to the fact that the smaller transmit power of the SU-Tx is allowed.

Figure 11 shows the dependence of the ergodic throughput on the interference threshold *Q_p_*. System performances are analyzed for different values of the transmission power of the PB and the outage probability of primary network *P*_out,PU_. 

With a high value of the interference threshold, saturation occurs for smaller values the of transmission power of the PB, while for larger values of transmission power of the PB, the ergodic throughput enters the saturation region.

For a higher interference threshold, the ergodic throughput begins to depend on the energy harvested, which is smaller for lower values of the PB’s transmission power. On the other hand, the ergodic throughput is independent of the PB’s transmission power for lower values of the interference threshold. In that case, the transmission power on the SU-Tx depends on the limitations caused by harmful interference on the PU, so only the outage probability of the primary network has an influence on the results. A higher value of the ergodic throughput is achieved if the outage probability of the primary network *P*_out,PU_ is higher. This is because with a higher value of the interference threshold and a higher value of outage probability of the primary network, a higher transmission power is allowed for the secondary user, with a lenient condition of harmful interference on the primary receiver.

The dependence of the ergodic throughput of the interference threshold *Q_p_* is presented in Figure 12. The analysis was performed based on the varying values of the distance from the PB to the SU-Tx and the outage probability of the primary network *P*_out,PU_. 

For the interference threshold lower than −5 dB, it can be noted that the ergodic throughput is independent of the distance from the PB to the SU-Tx. In that case, the transmission power of the SU-Tx is limited by the condition of harmful interference on the PU-Rx and depends on the interference threshold and outage probability of the primary network. When increasing the interference threshold to above −5 dB, less ergodic throughput is achieved if the distance from the PB to the SU-Tx is higher because in that case, the harvested energy at the SU-Tx, is smaller and less transmission power on the SU-Tx is available. With a higher value of the interference threshold and a higher value of the outage probability of the primary network, a larger transmission power is allowed on the SU, so higher values of the ergodic throughput are achieved. In the case in which the distance from the power beacon to the secondary transmitter is 10 m, saturation occurs when the interference threshold crosses a threshold approximately equal to 10 dB. In this case, the transmission power of the SU-Tx depends on the harvested energy, which is smaller when there is an increasing distance between the PB and the SU-Tx.

In Figure 13, the dependence of the ergodic throughput on the distance from the PB to the SU-Tx, *D_PB_*, the maximum distance from the secondary transmitter to the secondary receiver *D*, and the interference threshold *Q_p_* are presented. 

A smaller ergodic throughput is achieved if the maximum distance from the SU-Tx to the SU-Rx is higher because in this case, the received power at the SU-Rx is smaller. As in Figure 12, the same behavior of the ergodic throughput dependence on the distance from the PB to the SU-Tx *D_PB_* can be noted. For an interference threshold lower than −5 dB, it can be concluded that the ergodic throughput is independent of the distance from the PB to the SU-Tx, while above that interference threshold value, the ergodic throughput decreases with an increasing distance from the PB to the SU-Tx until saturation occurs in cases in which that distance increases to 15 m and the interference threshold is above 5 dB.

The dependence of the ergodic throughput on the time-switching factor is shown in Figure 14. System performances are analyzed for different values of the mean channel power gain in the Nakagami-*m* channel from the PB to the SU-Tx, a fixed value of the power of the PB, *P_PB_* = 20 dB, and different mobility models used for the description of the SU-Rx movement. The outage probability of the primary network is *P*_out,PU_ = 0.1, the mean channel power gain in the channel from the SU-Tx to the PU is Λ*_S_* = 1, the distance from the PB to the SU-Tx is *D_PB_* = 10 m, and the maximum distance from the SU-Tx to the SU-Rx is *D* = 5 m.

The ergodic throughput of the secondary network grows to some value of the time-switching factor; after this point, it decreases. It is possible to determine the peak of the ergodic throughput and the optimal value of the time-switching factor. If the mean channel power gain in the Nakagami-*m* channel from the PB to the SU-Rx is 10 dB, the peak of the ergodic throughput is achieved when the value of the time-switching factor is around 0.25 for each of the mobility models applied to the SU-Rx. The highest peak is reached by the 1D mobility model, which achieves better results than the other models. In the case in which the mean channel power gain in the Nakagami-*m* channel from the PB to the SU-Tx has a higher value, the peak of the ergodic throughput is reached for smaller values of the time-switching factor. The ergodic throughput is larger if the mean channel power gain in the Nakagami-*m* channel from the PB to the SU-Tx is higher, but increasing the time-switching factor leads to ergodic throughput independence on the mean channel power gain in the Nakagami-*m* channel from the PB to the SU-Tx.

## 6. Conclusions 

In this paper, a power-beacon-assisted cognitive system is analyzed based on spectrum sharing and available statistical CSI for the scenario in which the SU-Tx has a stationary position and the SU-Rx node is mobile. System performances are analyzed, and novel closed-form expressions are derived for the outage probability, the outage throughput, and the ergodic capacity. The theoretical expressions are verified using an independent simulation method.

Based on the obtained results, the influence of the interference limitation imposed by the primary network is discussed. The performance of the observed cognitive system depends on both PU system limitations, as well as the transmit power of the PB, the distances between the network nodes, and the channel power gain values. The best system performances are obtained when the permitted outage probability of the PU network and the required interference threshold have higher values. It has been observed that for very small values of the interference threshold *Q_p_*, system performances are independent of the PB’s power and the distance from the PB to the SU-Tx, as the limitation caused by the permitted interference on the PU-Rx is dominant with respect to the harvested energy. Furthermore, in the upper limit case, in which the interference threshold *Q_p_* has a great value, the saturation effect occurs as the system performance depends dominantly on the energy harvested from the PB. The provided analysis of the considered spectrally efficient system provides important guidelines for the design of future networks and IoT scenarios in which a higher presence of receiver mobility is expected. This analysis also represents the basis for the optimization of system parameters for various scenarios. Finally, as network nodes are computationally and energy constrained, future work will investigate issues related to the physical layer security of the analyzed system.

## Figures and Tables

**Table 1:** Summary of the literature.

Reference	Performances	WPT	CR	CSI	Fading Model	Mobility
[24]	Outage probability/average BER/throughput	+	−	−	Nakagami−m	RWP
[26]	Outage probability	+	+	−	Rayleigh	−
[27]	Probability density of the received power	−	−	−	−	RWP/RD
[31]	Outage probability/average BER	−	−	−	Nakagami−m; Nakagami−q (Hoyt), Rayleigh, and one−sided Gaussian distribution	RWP
[32]	Outage probability/average BER	−	−	−	Nakagami−m	RWP
[33]	Rate of IoT−enabled devices	+	−	−	Rayleigh	−
[34]	Outage probability/system throughput	+	−	−	Rayleigh	−
[35]	Outage probability	+	+	Imperfect	Rayleigh	−
[36]	Outage probability	+	+	Instantaneous	Nakagami−m	−
[37]	ergodic channel capacity	−	−	−	Generalized fading	RWP
[38]	Outage probability	−	−	−	*k* − *μ*	RWP
[39]	Outage probability/average BER	−	−	−	Rician fading	RWP
[40]	Outage probability/average BER	−	−	−	α−μ generalized fading	RWP
[41]	average error rate/average channel capacity	−	−	−	*κ* − *µ*	RWP
[42]	Outage probability	−	−	−	Nakagami−m	RD
[43]	Outage probability	−	−	−	Rayleigh	RD
[44]	Outage probability	−	−	−	Rayleigh	RWP/RD
[45]	Outage probability/average BER	−	+	imperfect	Rayleigh	RWP
[46]	Outage probability, average delay−limited throughput, ergodic capacity, and average delay−tolerant throughput	+	−	−	Nakagami−m	RWP
[47]	Achievable throughput	+	−	+	Nakagami−m	−
[48]	Ergodic capacity	+	−	Absence/partial	Rician	−
This paper	Outage probability/achievable throughput/ergodic throughput	+	+	Statistical	Nakagami−m	RWP

**Table 2:** Table of symbols.

Symbol	Explanation
*P_PB_*	the power of the signal from the PB
$P_{SU-Tx,max}^{}$	the maximal transmit power of the SU-Tx permitted by the PU
$P_{SU-Tx,EH}^{}$	the maximal transmit power of the SU-Tx enabled by EH
$E_{H}$	the energy harvested at the SU-Tx
*η*	the energy conversion efficiency coefficient
*T*	the time frame interval
α	the time-splitting factor
*P_out,PU_*	the maximal tolerable outage probability of the primary network
*k_S_*	the coefficient of adjustment of the SU-Tx power
*Q_p_*	the peak interference threshold of the PU
*h_PB_*	the fading envelope in the channel from the PB to the SU-Tx
$h_{TR}$	the fading envelope in the channel from the SU-Tx to the SU-Rx
*g_S_*	the fading envelope in the channel from the SU-Tx to the PU-Rx
$\gamma_{1}$	the instantaneous channel power gain in the channel from the PB to the SU-Tx
$\gamma_{2}$	the instantaneous channel power gain in the link from the SU-Tx to the SU-Rx
$\Lambda_{S}$	the average channel power gain in the link from the SU-Tx to the PU-Rx
*n_SU-Tx_*	the AWGN component at the SU-Tx
*x_PB_*	the energy signal sent from the PB
x*_SU-Tx_*	the information signal sent from the SU-Tx
$y_{SU-Tx}$	the received energy signal at the SU-Tx node
$y_{SU-Rx}$	the received information signal at the SU-Rx node
*n_SU-Rx_*	the AWGN component at the SU-Rx
*γ*	the instantaneous signal-to-noise ratio at the SU-Rx
$\sigma_{R}^{2}$	the mean power value of the AWGN component at the SU-Rx
*B_l_*, *β_l_*	the coefficients that correspond to topologies of the SU movement in 1D, 2D, or 3D
δ	the path loss exponent
*D_PB_*	the distance from the PB to the SU-Tx
*r*	the distance between the SU-Tx and the SU-Rx
*D*	the maximal distance between the SU-Tx and the SU-Rx
*m*_1_	Nakagami-*m* fading parameter in the channel from the PB to the SU-Tx
*m*_2_	Nakagami-*m* fading parameter in the channel from the SU-Tx to the SU-Rx
$\Omega_{1}$	the average channel power gain in the channel from the PB to the SU-Tx
$\Omega_{2}$	the average channel power gain in the channel from the SU-Tx to the SU-Rx
*γ_th_*	the outage threshold

**Table 3:** Polynomial coefficients.

Dimension	*n*	*B_l_*	*β_l_*
1	2	[6, −6]	[1, 2]
2	3	(1/73) [324, −420, 96]	[1, 3, 5]
3	3	(1/72) [735, −1190, 455]	[2, 4, 6]

**Table 4:** Simulation parameters.

Parameter	Figure 4	Figure 5	Figure 6	Figure 7	Figure 8	Figure 9	Figure 10	Figure 11	Figure 12	Figure 13	Figure 14
*P_out,PU_*	0.01	0.01–0.1	0.1	0.1	0.01	0.01	0.05	0.01–0.1	0.01–0.1	0.05	0.1
D*_PB_* [m]	1–10	1	10	5	1	1	5	10	5–10	5–15	10
D [m]	2–10	5	5–10	5	5–10	10	1–10	5	5	5–10	5
*Q_p_* [dB]	15	−20 ÷ 20	15	−5 ÷ 25	−20 ÷ 20	−20 ÷ 20	0 ÷ 20	−10 ÷ 15	−20 ÷ 15	−10 ÷ 12	15
P*_PB_* [dB]	30	20	20–30	20–30	30	30	20	20–30	30	30	20
Ω_1_ [dB]	10	10	20	20	10	10	10	20	10	10	20–30
Ω*_TR_* [dB]	30	20	20	20	30	30	20	20	30	30	20
Λ_S_	0.1–1	0.1	1	0.1–1	0.1	0.1–1	0.5–1	0.1	0.5	0.5	1
*γ_th_* [dB]	−5	5	5	−5	5	5	-	-	-	-	-
*η*	0.9	0.9	0.9	0.9	0.9	0.9	0.9	0.9	0.9	0.9	0.9
*α*	0.5	0.2	0.1–0.99	0.5	0.2	0.2	0.5	0.5	0.2	0.2	0.1–0.99
m_1_	1	1	1	1	1	1	1	1	1	1	1
m_2_	1	1	1	1	1–3	1–3	1	1	1	1	1
RWP	1D	1D–3D	1D	2D	1D	1D	2D	2D	2D	2D	1D–3D

## Appendix A

In order to obtain the solution for the integral *I*_1_ in the closed form, we apply following transformations. By using ([55], (1.211-1)) and with the help of ([56], (07.20.26.0006.01)), the integral *I*_1_ can be written as

(A1)
$$\begin{array}{ll} I_{1} & =\frac{\Gamma \left(1+m_{2}+\frac{\beta_{i}+1}{\delta }\right)}{\Gamma \left(m_{2}+\frac{\beta_{i}+1}{\delta }\right)}\sum_{k=0}^{\infty }\frac{1}{k!}{\left(-\frac{m_{1}}{\Omega_{1}}\right)}^{k}\\ & \times \int_{0}^{Q}G_{1,2}^{1,1}\left(\frac{m_{2}u}{wK_{1}\Omega_{2}}|\begin{array}{c} 1-m_{2}-\frac{\beta_{i}+1}{\delta }\\ \begin{array}{cc} 0, & -m_{2}-\frac{\beta_{i}+1}{\delta } \end{array} \end{array}\right)w^{m_{1}-m_{2}+k-1}dw. \end{array}$$

By applying transformation ([56], (07.34.17.0012.01)), we further achieve

(A2) $$G_{1,2}^{1,1}\left(\frac{m_{2}u}{wK_{1}\Omega_{2}}|\begin{array}{c} 1-m_{2}-\frac{\beta_{i}+1}{\delta }\\ \begin{array}{cc} 0, & -m_{2}-\frac{\beta_{i}+1}{\delta } \end{array} \end{array}\right)=G_{2,1}^{1,1}\left(\frac{wK_{1}\Omega_{2}}{m_{2}u}|\begin{array}{c} \begin{array}{cc} 1, & 1+m_{2}+\frac{\beta_{i}+1}{\delta } \end{array}\\ m_{2}+\frac{\beta_{i}+1}{\delta } \end{array}\right),$$

thus, the integral *I*_1_ can be written in following form:

(A3) $$\begin{array}{ll} I_{1} & =\frac{\Gamma \left(1+m_{2}+\frac{\beta_{i}+1}{\delta }\right)}{\Gamma \left(m_{2}+\frac{\beta_{i}+1}{\delta }\right)}\sum_{k=0}^{\infty }\frac{1}{k!}{\left(-\frac{m_{1}}{\Omega_{1}}\right)}^{k}\\ & \times \int_{0}^{Q}G_{2,1}^{1,1}\left(\frac{wK_{1}\Omega_{2}}{m_{2}u}|\begin{array}{c} \begin{array}{cc} 1, & 1+m_{2}+\frac{\beta_{i}+1}{\delta } \end{array}\\ m_{2}+\frac{\beta_{i}+1}{\delta } \end{array}\right)w^{m_{1}-m_{2}+k-1}dw, \end{array}$$

which can finally be solved by using transformation ([56], (07.34.21.0085.01))

(A4) $$\begin{array}{ll} I_{1} & =\left(m_{2}+\frac{\beta_{i}+1}{\delta }\right)\sum_{k=0}^{\infty }\frac{1}{k!}{\left(-\frac{m_{1}}{\Omega_{1}}\right)}^{k}\\ & \times Q^{m_{1}-m_{2}+k}G_{3,2}^{1,2}(\frac{K_{1}\Omega_{2}Q}{m_{2}u}|\begin{array}{c} \begin{array}{ccc} 1-m_{1}+m_{2}-k, & 1, & 1+m_{2}+\frac{\beta_{i}+1}{\delta } \end{array}\\ \begin{array}{cc} m_{2}+\frac{\beta_{i}+1}{\delta }, & m_{2}-m_{1}-k \end{array} \end{array}). \end{array}$$

The solution of the integral *I*_2_ can be obtained by transformation $x=\frac{m_{2}}{\Omega_{2}}w$ and applying ([55], (8.350.2)), when we obtain

(A5) $$I_{2}={\left(\frac{\Omega_{1}}{m_{1}}\right)}^{m_{1}}\Gamma \left(m_{1},\frac{m_{1}}{\Omega_{1}}Q\right).$$

## Appendix B

In order to obtain the solution for the integral *I*_3_, we apply the transformation of the MeijerG-function provided by ([56], (07.34.17.0012.01)):

(A6) $$I_{3}=\int_{0}^{\gamma_{th}}u^{m_{2}-1}G_{2,3}^{2,1}\left(\frac{m_{2}u}{K_{1}\Omega_{2}Q}|\begin{array}{c} \begin{array}{cc} 1-m_{2}-\frac{\beta_{i}+1}{\delta }, & 1-m_{2}+m_{1}+k \end{array}\\ \begin{array}{ccc} m_{1}-m_{2}+k, & 0, & -m_{2}-\frac{\beta_{i}+1}{\delta } \end{array} \end{array}\right)du.$$

Then, by using ([56], (07.34.21.0084.01)), the integral *I*_3_ is solved in the exact closed form:

(A7) $$I_{3}=\gamma_{th}^{m_{2}}G_{3,4}^{2,2}\left(\frac{m_{2}\gamma_{th}}{K_{1}\Omega_{2}Q}|\begin{array}{c} \begin{array}{ccc} 1-m_{2}, & 1-m_{2}-\frac{\beta_{i}+1}{\delta }, & 1-m_{2}+m_{1}+k \end{array}\\ \begin{array}{cccc} m_{1}-m_{2}+k, & 0, & -m_{2}-\frac{\beta_{i}+1}{\delta }, & -m_{2} \end{array} \end{array}\right).$$

We are solving integral *I*_4_ by transforming the hypergeometric function into a Meijer G-function using equation ([56], (07.20.26.0006.01))

(A8) $$I_{4}=\frac{\Gamma \left(1+m_{2}+\frac{\beta_{i}+1}{\delta }\right)}{\Gamma \left(m_{2}+\frac{\beta_{i}+1}{\delta }\right)}\int_{0}^{\gamma_{th}}u^{m_{2}-1}G_{1,2}^{1,1}\left(\frac{m_{2}u}{K_{2}\Omega_{2}}|\begin{array}{c} 1-m_{2}-\frac{\beta_{i}+1}{\delta }\\ \begin{array}{cc} 0, & -m_{2}-\frac{\beta_{i}+1}{\delta } \end{array} \end{array}\right)du,$$

while the final expression is obtained by using identity ([56], (07.34.21.0084.01))

(A9) $$I_{4}=\left(m_{2}+\frac{\beta_{i}+1}{\delta }\right)\gamma_{th}^{m_{2}}G_{2,3}^{1,2}\left(\frac{m_{2}\gamma_{th}}{K_{2}\Omega_{2}}|\begin{array}{c} \begin{array}{cc} 1-m_{2}, & 1-m_{2}-\frac{\beta_{i}+1}{\delta } \end{array}\\ \begin{array}{ccc} 0, & -m_{2}-\frac{\beta_{i}+1}{\delta }, & -m_{2} \end{array} \end{array}\right).$$

## Appendix C

In (27), we express the logarithm function as a Meijer G-function, using ([56], (01.04.26.0003.01)) as

(A10) $$\ln\left(1+u\right)=G_{2,2}^{1,2}\left(u|\begin{array}{c} 1,1\\ 1,0 \end{array}\right).$$

Based on the previously derived closed-form Equation (19) for the PDF of the SNR at the secondary receiver, we transform the argument of the Meijer G-function in the PDF expression using ([56], (07.34.16.0002.01)), and we express the hypergeometric function in the PDF expression using the Meijer G-function, as in ([56], (07.20.26.0006.01)). Thus, the obtained PDF expression can be written in the following form:

(A11) $$\begin{array}{ll} f_{\gamma }\left(u\right) & =C\frac{u^{m_{2}-1}}{K_{1}^{m_{2}}}\sum_{i=1}^{n}\sum_{k=0}^{\infty }\frac{1}{k!}{\left(-\frac{m_{1}}{\Omega_{1}}\right)}^{k}B_{i}\frac{\left(m_{2}+\frac{\beta_{i}+1}{\delta }\right)}{m_{2}\delta +\beta_{i}+1}\;\\ & \times Q^{m_{1}-m_{2}+k}G_{2,3}^{2,1}\left(\frac{m_{2}u}{K_{1}\Omega_{2}Q}|\begin{array}{c} \begin{array}{cc} 1-m_{2}-\frac{\beta_{i}+1}{\delta }, & 1-m_{2}+m_{1}+k \end{array}\\ \begin{array}{ccc} m_{1}-m_{2}+k, & 0, & -m_{2}-\frac{\beta_{i}+1}{\delta } \end{array} \end{array}\right)\\ & +C\frac{u^{m_{2}-1}}{K_{2}^{m_{2}}}{\left(\frac{\Omega_{1}}{m_{1}}\right)}^{m_{1}}\Gamma \left(m_{1},\frac{m_{1}}{\Omega_{1}}Q\right)\\ & \times \sum_{i=1}^{n}\frac{B_{i}}{m_{2}\delta +\beta_{i}+1}\left(m_{2}+\frac{\beta_{i}+1}{\delta }\right)G_{1,2}^{1,1}\left(\frac{m_{2}u}{K_{2}\Omega_{2}}|\begin{array}{c} 1-m_{2}-\frac{\beta_{i}+1}{\delta }\\ \begin{array}{cc} 0, & -m_{2}-\frac{\beta_{i}+1}{\delta } \end{array} \end{array}\right)\;. \end{array}$$

By replacing previous Equations (A10) and (A11) in (27), we obtain

(A12) $$\begin{array}{ll} C_{\mathrm{erg}} & =\frac{1}{\ln2}\frac{C}{K_{1}^{m_{2}}}\sum_{i=1}^{n}\sum_{k=0}^{\infty }\frac{1}{k!}{\left(-\frac{m_{1}}{\Omega_{1}}\right)}^{k}B_{i}\frac{\left(m_{2}+\frac{\beta_{i}+1}{\delta }\right)}{m_{2}\delta +\beta_{i}+1}\;Q^{m_{1}-m_{2}+k}\\ & \times \int_{0}^{\infty }u^{m_{2}-1}G_{2,2}^{1,2}\left(u|\begin{array}{c} 1,1\\ 1,0 \end{array}\right)G_{2,3}^{2,1}\left(\frac{m_{2}u}{K_{1}\Omega_{2}Q}|\begin{array}{c} \begin{array}{cc} 1-m_{2}-\frac{\beta_{i}+1}{\delta }, & 1-m_{2}+m_{1}+k \end{array}\\ \begin{array}{ccc} m_{1}-m_{2}+k, & 0, & -m_{2}-\frac{\beta_{i}+1}{\delta } \end{array} \end{array}\right)du\\ & +\frac{1}{\ln2}\frac{C}{K_{2}^{m_{2}}}{\left(\frac{\Omega_{1}}{m_{1}}\right)}^{m_{1}}\Gamma \left(m_{1},\frac{m_{1}}{\Omega_{1}}Q\right)\sum_{i=1}^{n}B_{i}\frac{\left(m_{2}+\frac{\beta_{i}+1}{\delta }\right)}{m_{2}\delta +\beta_{i}+1}\\ & \times \int_{0}^{\infty }u^{m_{2}-1}G_{2,2}^{1,2}\left(u|\begin{array}{c} 1,1\\ 1,0 \end{array}\right)G_{1,2}^{1,1}\left(\frac{m_{2}u}{K_{2}\Omega_{2}}|\begin{array}{c} 1-m_{2}-\frac{\beta_{i}+1}{\delta }\\ \begin{array}{cc} 0, & -m_{2}-\frac{\beta_{i}+1}{\delta } \end{array} \end{array}\right)du. \end{array}$$

Finally, using ([56], (07.34.21.0011.01)) on both integrals, we obtain the closed-form expression provided by (29).
